# Evaluation of Agility and Walking Ability in Elderly People

**DOI:** 10.1093/geroni/igaa057.651

**Published:** 2020-12-16

**Authors:** Ken Yamauchi, Hiromichi Hasegawa, Akira Ogita, Tutomu Ichikawa, Shota Kagawa, Yamauchi Ken

**Affiliations:** 1 Keio University, Yokohama, Kanagawa, Japan; 2 NPFC, Nagakute, Aichi, Japan; 3 Osaka City University, Osaka, Japan; 4 Matsuyama Shinonome Junior College, Matsuyama, Ehime, Japan; 5 Tohto University, Chiba-shi, Chiba, Japan

## Abstract

An important aspect of independent living in a super-aging society is the achievement of “successful aging” in mind and body. Recent large-scale epidemiological studies found that reduced walking ability significantly affects “extended healthy life expectancy” and “successful aging.” In terms of anatomy and physiology, “usual aging” (i.e., the inevitable decrease in walking function due to aging) occurs unless one undertakes daily physical activity. Conversely, aggressive moderate and habitual physical activity may lead to “successful aging” (i.e., maintained and improved walking function). Previous studies revealed that agility and walking ability are related to healthy life expectancy. Thus, we measured agility with a stick response test to investigate its relationship to gait, and determined whether this test could be substituted for a battery test of walking ability. This test has the convenience of shortening measurement time and requiring a wider measurement place than gait evaluation performed by actually walking. Correlation was observed between the rod response test and stride length (r = 0.60), walking speed (r = 0.38), and walking ratio (r = 0.34). Therefore, we propose that the rod response measurement is simpler than a simple walking ability test and can be used as a physical function measurement item that predicts the health and longevity of the elderly.

